# New Paradigm in Cell Therapy Using Sperm Head to Restore Brain Function and Structure in Animal Model of Alzheimer's Disease: Support for Boosting Constructive Inflammation *vs*. Anti-Inflammatory Approach

**DOI:** 10.1155/2022/8343763

**Published:** 2022-04-07

**Authors:** Nafiseh Pakravan, Ardeshir Abbasi, Zuhair Mohammad Hassan

**Affiliations:** ^1^Division of Immunology, Medical School, Alborz University of Medical Sciences, Karaj, Iran; ^2^Department of Immunology, Faculty of Medical Sciences, Tarbiat Modares University, Tehran, Iran

## Abstract

Alzheimer's is characterized by accumulation of amyloid-*β* (A*β*) associated with insufficient clearance of toxicants from the brain establishing a chronic inflammation and other abnormalities in the brain. Inflammatory microglia and astrocytes along with abnormal lymphatics associated with insufficient clearance of A*β* and other toxicants from the brain establish a chronic inflammation. This causes abnormal choroid plexus, leukocyte trafficking, and hypoxic condition along with high levels of regulatory T cells (Tregs). There is no consensus among researchers regarding decreasing or increasing Tregs to achieve therapeutic effects. Different opposing studies tried to suppress or boost inflammation to treat AD. Based on reproductive immunology, sperm induces constructive inflammatory response and seminal-vesicle-fluid (SVF) suppresses inflammation leading to uterus remodeling. It prompted us to compare therapeutic efficiency of inflammatory or anti-inflammatory approaches in AD model based on reproductive immunology. To do so, SVF, sperm, or sperm head (from Wistar rat) was administered via intra-cerebro-ventricular route to Sprague Dawley rat AD model. Behavioral and histological examination were made and treatment groups were compared with control AD model and normal groups. Therapeutic efficacy was in the order of sperm head>sperm>SVF. Sperm head returned learning memory, A*β*, lymphatics, neural growth factors, choroid plexus function, Iba-1/GFAP, MHC II/CD86/CD40, CD38/IL-10, and hypoxia levels back to normal level. However, SVF just partially ameliorated the disease. Immunologic properties of sperm/sperm head to elicit constructive inflammation can be extended to organs other than reproductive. This nature-based approach overcomes genetic difference as an important obstacle and limitation in cell therapy, and is expected to be safe or with least side effects.

## 1. Introduction

Alzheimer's disease (AD) was for the first time diagnosed and described by Alois Alzheimer. The disease is one of the most common types of dementia due to accumulation of senile plaques made up of amyloid-*β* (A*β*) plaques and neurofibrillary tangles composed of hyperphosphorylated tau protein [1]. A*β* plaques are formed following impaired clearance of A*β* and other toxicants from the brain which has been attributed to abnormality in the lymphatics draining the brain [2, 3].

In addition, as the central nervous system (CNS) resident cells and main responders to homeostasis interrupters, microglia and astrocytes accumulate around the senile plaques and become inflammatory to remove the plaques but fail [4]. This helps maintenance of chronic inflammation within CNS.

Microglia's role during an inflammatory response may exhibit opposing activation status including beneficial or detrimental. In the beneficial case, activated microglia produce a wide array of neuroprotective and neural growth factors preventing neuronal injury ending up with resolution of inflammation. Inversely in the detrimental case, inflammatory mediators released by microglia disturb neuronal functions and cause damage by continuing inflammatory process [4]. Depending on the microenvironment and milieu, microglia can be polarized into a proinflammatory (M1) or an anti-inflammatory (M2) activation status to adapt to their microenvironment [5]. Astrocytes are one of the microenvironmental factors which have the capacity to cross talk with microglia affecting their activation status [6, 7]. During neuroinflammation, astrocytes along with microglia participate in the secretion of inflammatory mediators playing a crucial role in the regulation of the ongoing inflammatory process in AD and its animal model [8]. Inflammatory microglia and astrocytes have cross talk with brain Tregs interacting with each other and create a hypoxic condition [9, 10].

The chronic inflammatory condition is also associated with dysfunction of choroid plexus gateway leading to dysregulated trafficking of immune cells and notably increase of Tregs. This follows neuronal damage, brain tissue destruction, and consequently gradual loss of cognitive function [1, 11, 12]. The orchestration among abnormal lymphatics, choroid plexus gateway function, hypoxia, and insufficient levels of growth factors make the disease more complicated to treat [12–15].

The immune system has important role in the development, health, damage, and repair and regeneration of the CNS [16]. Considering the inflammatory nature of AD, immunotherapeutic approaches look as plausible final solution. In general, current immunotherapies suggested for AD treatment are based on active or passive targeting A*β*/tau or immunosuppression, none of which has led to recovery [17–19]. Alternatively, a new method based on boosting inflammation has also been proposed which is not applicable in clinic because of potential development of autoimmunity [20]. Effectiveness of opposing therapeutic strategies and multi-factorial nature of the disease shows that the final solution demands more elaborate and clever approach. Development of new approaches inspired by nature associated with safety looks plausible.

Immunologic modification during mating makes a fundamental rearrangement throughout the body persisting during pregnancy. The changes commence in the vicinity of the uterus and also extend to far areas of the body such as the brain, though in lower magnitude. As an example, autoimmune attack in multiple sclerosis is ameliorated during pregnancy [21, 22]. Studies on reproductive immunology have shown that seminal vesicle fluid (SVF) has anti-inflammatory property and increases Tregs [22–24]. In contrast, sperm/sperm head promotes a constructive inflammatory response by recruitment and programming of neutrophils leading to elimination of debridement and tissue remodeling and reconstitution in the uterus during mating suitable for the fetus [25–27]. To examine and compare both of anti-inflammatory and inflammatory approaches, we attempted to simulate the immune response in the reproductive system and developed it in organs other than reproductive. Since the brain has low regenerative capacity, we chose damaged brain in animal model of AD [28]. Therefore, with the aim of devising a nature-based approach of suppressing inflammation (by SVF) or boosting constructive inflammatory response (by sperm), SVF and sperm were administered in an animal model of AD. It is noteworthy to re-mention that the magnitude of immunological changes during mating in other organs depends on the distance from the reproductive organ, the farther the distance the lower the changes [22]. On this basis, treatments were performed via intra-cerebro-ventricular (ICV) route.

## 2. Materials and Methods

### 2.1. Ethics Statement and Study Protocol

Ethical Committee of the Alborz University of Medical Sciences approved the project under reference No. Abzums.Rec.1395.11. Animal Care and Use Protocol of Alborz University of Medical Sciences in compliance with Ethical Committee of Iran Ministry of Health and Education were considered during animal care or treatments.

### 2.2. Animals

Adult male Sprague–Dawley rats weighed 300–320 g at the time of surgery obtained from Razi Institute of Iran, Karaj, Iran. Animals were randomly grouped, housed, and acclimatized to laboratory conditions prior to experimentation under standard conditions of 12 h light and dark cycle with food and water ad libitum.

### 2.3. Preparation of A*β*_1–42_-Induced AD Rat Model

The animal model of AD was set up using A*β*_1–42_ (Sigma-Aldrich, St. Louis, Missouri, USA) by incubating it in sterile saline to a final concentration of 1 mg/ml at 37°C for 7 days [29]. This allowed the peptide to fibrillize and aggregate which are associated with toxicity and stored at -20°C. The A*β*_1–42_ solution generally containing insoluble precipitates of both fibril-like structures and different-sized oligomers facilitates markedly learning deficits. To inject A*β*_1–42_ solution, rats were first deeply anesthetized by intraperitoneal injection of ketamine (70 mg/kg, Interchemie, Netherland)/xyline 10 mg/kg, Bremer/pharmaGmBH, Germany). The animals were then placed in a stereotactic apparatus (RWD 68505, Brazil), the skull was positioned in the frame, and a midline sagittal incision was made along the midline. The area surrounding bregma was cleaned and dried and the incubated A*β*_1–42_ solution was injected into the dentate gyrus region of rat hippocampus with a volume of 5 *μ*l containing 5 *μ*g _A*β*1–42_ very slowly (over 2 min) bilaterally. Stereotaxic injection coordinates were as anterior-posterior (AP), −3.3 mm; medial-lateral (ML), +-1.8 mm; ventral (V), −2.5 mm; by measuring the distances from bregma. To prevent A*β*_1–42_ reflux out of the needle tract and maximize diffusion, the needle remained at the injection site for 1 min after injection. The area was then closed using a suture. Penicillin (100,000 U) was injected to each rat in the hindquarter muscle to prevent post-surgery infection.

### 2.4. ICV Administration of Seminal Vesicle Fluid (SVF), Sperm, or Sperm Head

To estimate therapeutic efficacy of SVF, sperm, or sperm head, the animals were divided into six groups (*n* =6) including untreated AD model, saline-treated AD model, SVF-treated AD model, sperm-treated AD model, sperm head-treated AD model, and normal. Untreated control and saline-treated control animals were both used as diseased control. Normal healthy animals were also used as healthy control including intact normal and saline-treated normal animals. The results between the groups were not different and the results presented in this article are related to saline-treated normal animals. In addition, the results between the two groups of AD model without treatment and saline-treated AD model were similar. Therefore, just results of untreated control AD model are presented throughout the manuscript. In addition, normal animals receiving saline did not develop pathological signs observed in the AD model developed by intra-hippocampal injection of A*β* in saline.

To prepare sperm head, a pool of sperm was prepared from five Wistar rats and sperm head was prepared following three steps of centrifuging and using ficoll and SVF was prepared from mature male rats as described before [23, 24]. Sperm and SVF were prepared from Wistar rats to make sure that this method overcomes genetic differences which is an important obstacle in stem cell therapy. In addition, immunologic properties of sperm and SVF naturally are even exerted in an allogenic manner.

Seven days after A*β*_1–42_ injection, saline, SVF, 10 × 10^6^ sperm, or 10 × 10^6^ sperm head was injected at the final volume of 25 *μ*l into the left and right lateral cerebral ventricle with the following coordinates: AP: -0.5 mm; ML: ±1.2 mm; DV: −3.2 mm. Sperm or sperm head was prepared from five rats and suspended in saline. Seven days following treatment via ICV route, the animals were sacrificed and behavioral, immunohistochemical, and molecular experiments were performed as described below.

### 2.5. Behavioral Testing: Morris Water Maze

Morris water maze test has been commonly applied for research on specific aspects of spatial learning was performed [29]. This approach is based on the belief that animals find a suitable strategy to search their environment and the risk (in this case of water) that they will get the favored result with minimal effort. Morris maze is a water tank with 180 cm diameter and 60 cm depth. About half of the tank is filled with water. The tank is divided into four hypothetical equal parts and an escape platform (25 cm height and 10 cm diameter) is placed in one of the four sections. The platform is 1-2 cm below the surface of the water and cannot be seen from the outside. The maze is located in a room containing various spatial symptoms which remain constant during experiments and the animals can see the symptoms during the test. A video camera mounted at a height of 180 cm overhead the center of the water maze monitors the collection and automatically records the information about the ongoing experiment by a video tracking system connected to the computer. The test includes two phases of acquisition and retention. The acquisition phase measures the animal's learning process based on the time elapsed and the distance traveled to find the escape platform. The retention phase assesses memory retention. For the prior habituation, the rats were taken to the tank one day before commencing the test.

During the acquisition phase, animals are trained four times for four sequential days with the help of several brightly colored signs visible from the pool. In each trial, animals were subjected to four sequential trials with a gap lasting 5 min. The animal was gently put between any one of the quadrant while facing the wall of pool, and then released. Maximum time for the animal to find the escape platform was 60 sec. Every animal that could not find the escape platform during this time would be guided to the platform and allowed to sit on it for 30 sec. The animal remembers its position due to the location of the platform and the cues installed in the laboratory during this 30 sec. The animals that could find the platform were given 30 sec to memorize the cues. On the fifth day and at the next phase, the animal was released randomly at one of the edges facing the wall of the tank to evaluate its memory retention. Time spent by animal in the hidden platform on day 5 was recorded as spending time in the target quadrant. EthoVision V7.1 automated tracking system (Noldus Information Technology) was used to record the data.

### 2.6. Tissue Preparation for Cresyl Violet and Immunohistochemical Staining

Seven days after ICV treatment, the animals were anesthetized using a ketamine and xylazine mixture, transcardially perfused with cold saline, and the brain was taken out. The dissected tissue was fixed in neutral buffered 10% formalin. This was followed by paraffin embedding and 5 *μ*m thick sections were prepared on a rotary microtome (Leica, Germany). The sections were placed on polylysine-coated slides and used for the immunohistochemical staining. Haematoxylin and eosin (H&E) staining was also done on tissue samples of 5 *μ*m thick sections prepared from serial sections and based on the common method in which the sections were initially rehydrated using differential alcohol gradients for subsequent H&E dyeing, dehydrated using grade series of alcohol, submerged in xylene, and mounted in neutral Entellan [29]. Evaluation of lymphatic vessels in H&E-stained tissue sections was performed by a blinded expert based on the criteria described before [30].

Cresyl violet staining was performed on the tissue sections by rehydrating in distilled water and soaking in 0.1% cresyl violet for about 20 min until the desired depth of staining was achieved. The tissue sections were rinsed in distilled water, dehydrated in graded serried of ethanol, immersed in xylene, and mounted in entellan. After being cover slipped, neuronal loss characterized as Nissl-positive cells was examined and assessed. The percentage of apoptotic cells was determined on cresyl violet-stained tissue sections. For counting the number of apoptotic cells, five random views in each section were measured per rat using a light microscope and the number of apoptotic cells was quantified by a vet pathologist with no knowledge of the experiment. Apoptotic cells were evaluated based on cell shrinkage, loss of uniformity of Nissl body, cytoplasm, nucleus density, and pyknotic nucleus. Viable cells were considered cells with orderly arranged cells with normal morphology, abundant cytoplasm, and evident nucleus and nucleolus [31, 32].

Immunohistochemical staining on the tissue sections of hippocampal, lymphatic vessel, and choroid plexus sections was performed [29]. The tissue sections were blocked with 0.3% Triton X-100 and 10% goat serum in PBS (pH 7.3) for 30 min and incubated overnight at room temperature with primary antibodies (1 : 100, Biorbyt, Cambridge, UK) including A*β*, Brain-Derived Neurotrophic Factor (BDNF), Chemokine (C-C motif) Ligand 21 (CCL21), Cluster of Designation 31 (CD31), CD38, CD40, C-X-C motif chemokine-10 **(**CXCL10), Glial Fibrillary Acidic Protein (GFAP), Hypoxia Inducible Factor-1*α* (HIF-1*α*), Intercellular Adhesion Molecule-1 (ICAM-1), Interleukin-10 (IL-10), Ionized calcium binding adaptor molecule-1 (Iba-1), Major Histocompatibility Complex II (MHC II), Nerve Growth Factor (NGF), and Vascular Cell Adhesion Molecule-1 (VCAM-1). After washing with 0.01 M PBS, the tissue sections were incubated with FITC conjugated donkey anti-rabbit IgG (Biorbyt, Cambridge, UK) diluted in 0.01 M phosphate buffer saline (PBS) (1 : 200) as the secondary antibody for 2 h at room temperature. Then, after rinsing with 0.01 M PBS, the sections were stuck to glass slides and observed using a fluorescence microscope. DAPI (4 ′-6-diamidino-2-phenylindole) was used for nuclei staining in each section and hematoxyline was used as counterstaining in immunohistochemical assessment. For each marker, the expression of green fluorescent was randomly examined in five microscopic fields with 400x magnification. Indeed, the total number of cells in a field was counted and the expression of the marker was presented as a percentage of the expression relative to the nucleus of the cells in that microscopic field. In order to evaluate the expression of A*β* plaques, the images were converted to black and white images using ImageJ software. The total amount of tissue in each microscopic field was determined as a percentage by measuring the number of pixels in the image. Then, the amount of beta-amyloid plaques was determined based on the percentage. Finally, the percentage of amyloid beta marker was divided into the total tissue visible in the image and presented as a percentage.

Triple immunohistochemical staining was also carried out for detection of Tregs utilizing CD4, CD25, and Forkhead box protein 3 (FoxP3) expressions. In the case of CD4 and FoxP3, anti-CD4 (1 : 500; Biolegend, London, UK) raised in mouse and FoxP3 (1 : 500; AVIVA System Biology, San Diego, US) raised in goat were used for staining of CD4 and FoxP3 in Tregs. CD25 expression was detected using rabbit antibody (1 : 100; Biorbyt, Cambridge, UK). After rinsing in PBS with 0.5% BSA, the sections were incubated with a mixture of secondary antibodies including FITC donkey anti-rabbit, PE donkey anti-mouse, and blue donkey anti-goat (1 : 200; Biolegend, London, UK; Abcam, Cambridge, UK; Abcam, Cambridge, UK). Then in each microscopic field (400x), total cells were counted in the image with ImageJ software. The expression ratio of the markers to the total cells in the image was evaluated. The expression of all three markers to all cells was reported as 100.

All of microscopic parameters and setting were identical during the experiments. To keep consistency within and among the different experimental cases, matched tissue sections were always processed. Quantification and analysis of the immunohistochemically stained tissue sections were performed after taking digitized images using a Zeiss Axioplan 2 fluorescent microscope. The five samples in each experimental group and five fields of each sample were used for capturing. Each microscopic field was visualized under a light microscope (Olympus, Japan). FITC expression was measured after subtracting the background value using ImageJ software (version: 1.52 h) based on nuclear staining, indicating positive reaction for the desired markers by an expert blinded to the origin of the sample.

### 2.7. RNA Extraction and Real-time PCR

The perivascular lymphatic vessel along superior sagittal sinus was separated from frozen brain and total RNA was extracted using CinnaPure RNA Purification Kit (Cinnagen, Karaj, Iran) according to the manufacturer's protocol. The quality and quantity of RNA concentrations were evaluated using Nano Drop 2000c (Eppendorf, Germany). Expression of mRNA for *β-actin*, *glyceraldehyde 3-phosphate dehydrogenase* (*GAPDH*), *lymphatic vessel endothelial hyaluronan receptor-1* (*LYVE-1*), *prospero-related homeobox1* (*Prox1*), *podoplanin* (*PDPN*), *vascular endothelial growth factor receptor3* (*VEGF-R3*), and *VEGF-C* was determined using ABI Step One (Applied Biosystems, Sequences Detection Systems. Foster City, CA) thermal cycler apparatus and SYBR® Green PCR master mix kit (Applied Biosystems, Life Technologies, Paisley, United Kingdom) according to the manufacturer's instructions. Each reaction contained 5 *μ*l master mix, 100 nM primers for reference gene, *LYVE-1*, *Prox1*, *PDPN*, *VEGF-R3*, and *VEGF-C* plus 1 *μ*g template cDNA. The sequences for primers were as forward 5′-ATCTGGCACCACACCTTC-3′ and reverse 5′-AGCCAGGTCCAGACGCA-3′ for *β-actin*, forward 5′-AAGTTCAACGGCACAGTCAAGG-3′ and reverse 5′-CATACTCAGCACCAGCATCACC-3′ for *GAPDH*, forward 5′- AGGTATGGATGGGTTGGAGA-3′ and reverse 5′- GGGGTTTGAGTGTTGAATGTGG-3′ for *LYVE-1*, forward 5′-GGTCTTCAGGAGAAGGGAATG-3′ and reverse 5′- GGTATTCCAGTGTGTGAAGTG-3′ for *Prox1*, forward 5′-GTTGGTCTGGGTTTTGGGG-3′ and reverse 5′-CAATGGGAGGCTGTGTTGGT-3′ for *PDPN*, forward 5′-TGGGCAGGAGGTGTTGTGGGA-3′ and reverse 5′-GAGGAAGGGATTGGAAAGGA-3′ for *VEGF-R3*, and forward 5′-GATGTGGGGAAGGAGTTTGGAG-3′ and reverse 5′-CTGATTGTGACTGGTTTGGGG-3′ for *VEGF-C*. The primer's efficiency and specificity, fidelity of real-time PCR, and melting curve analysis were controlled as described before [23, 24]. Thermocycler conditions commenced with an initial step at 95°C for 15 min. This was followed by 40 cycles at 94°C: 20 sec, 58-60°C: 40 sec, and 72°C: 30 sec. The *GAPDH* and *β*-actin genes were chosen as reference genes against which mRNA expression of the target gene was normalized. The result was analyzed using two reference genes in parallel to make sure of the validation of the housekeeping gene [33]. However, the results presented in this article are on the basis of *β*-actin. The resultant gene expression level was presented as 2^-*ΔΔ*Ct^, in which *Δ*Ct showed the difference between Ct values of target gene and reference gene [34].

### 2.8. Statistical Analysis

Statistical operations were performed using GraphPad Prism software (GraphPad Software, San Diego, CA) to analyze the data using one-way ANOVA to compare between groups followed by Tukey's post hoc test. The results of acquisition training of the Morris water maze were analyzed using two-way ANOVA and the Bonferroni post hoc procedure. All of the experiments were performed in triple. Differences were considered statistically significant when *p* value was less than 0.05.

## 3. Results

### 3.1. Evaluation of Spatial Learning Memory, Neural Apoptosis, and A*β* Plaque Burden after Administration of Seminal Vesicle Fluid (SVF), Sperm, or Sperm Head

**Figure 1:**
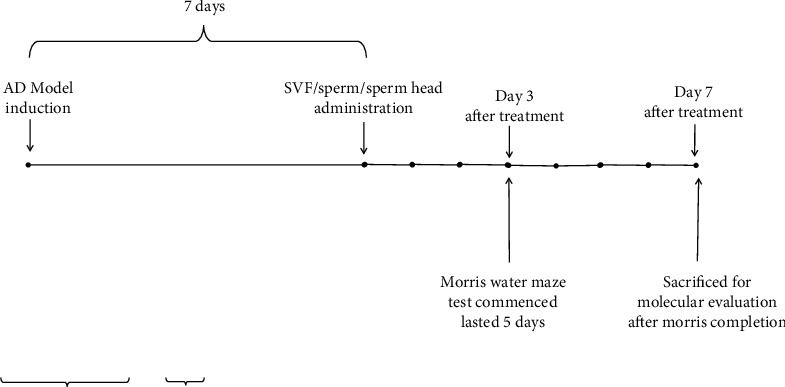


**Figure 2:**
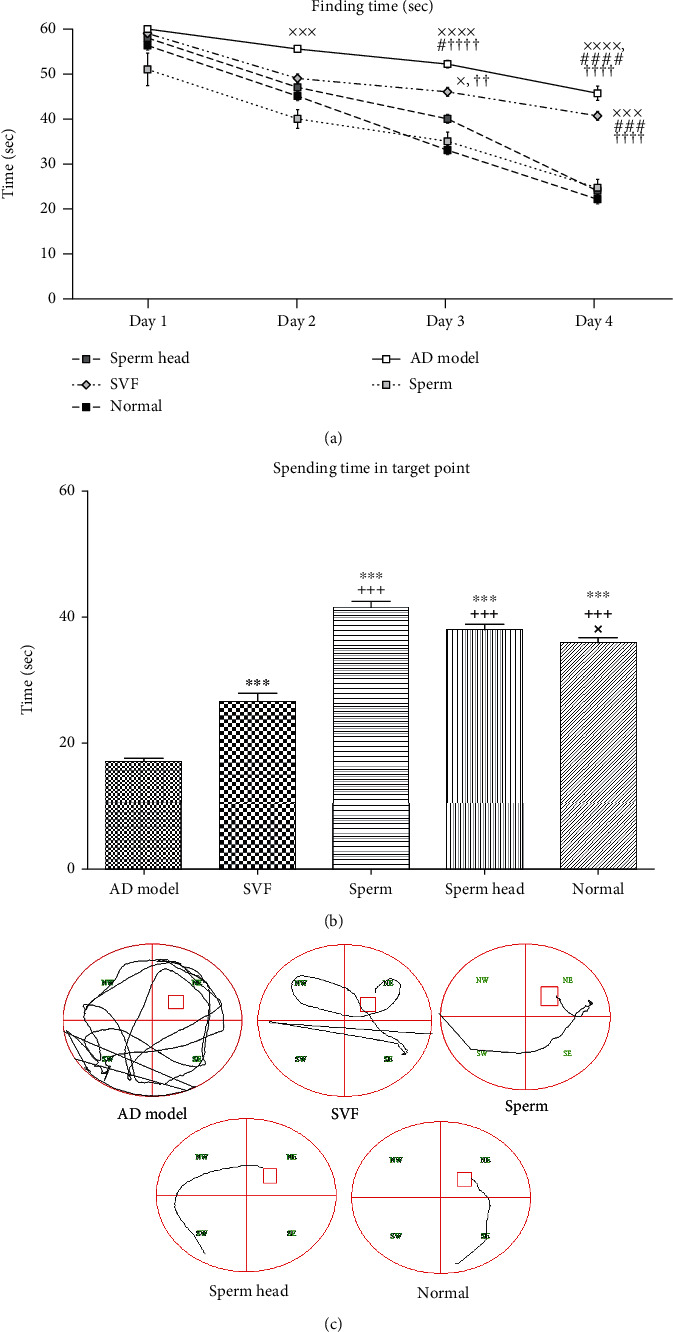


**Figure 3:**
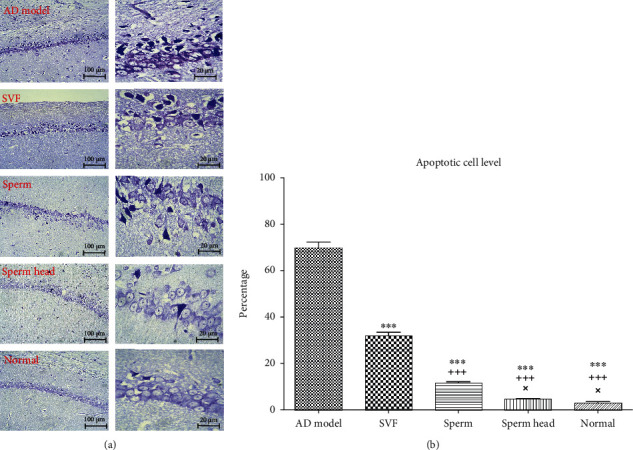


Based on our preliminary experiments, SVF, sperm, or sperm head was injected via ICV route and comparison was made with the control AD model and normal groups according to the plan presented in Figure 1. As Figures 2(a)–2(c) show, SVF did not significantly affect spatial learning memory but decreased the rate of apoptotic cells compared to the control AD model (*p* < 0.001, Figures 3(a) and 3(b)). However, the level of apoptotic cell in SVF-treated AD model was significantly higher than that of sperm- or sperm head-treated AD model animals (*p* < 0.001) and could not return the situation back to the normal state (*p* < 0.001). Sperm recovered spatial learning memory deficit comparing with the control AD model (*p* < 0.0001) and SVF-treated AD model (*p* < 0.001) and was efficiently neuroprotective by decreasing the rate of apoptotic cells compared to the control AD model (*p* < 0.001) and SVF-treated AD model (*p* < 0.001) albeit not at the level of the sperm head-treated AD model or normal animals (*p* < 0.05). There was no significant difference in spatial learning memory and neural cell survival between sperm head and normal group.

**Figure 4:**
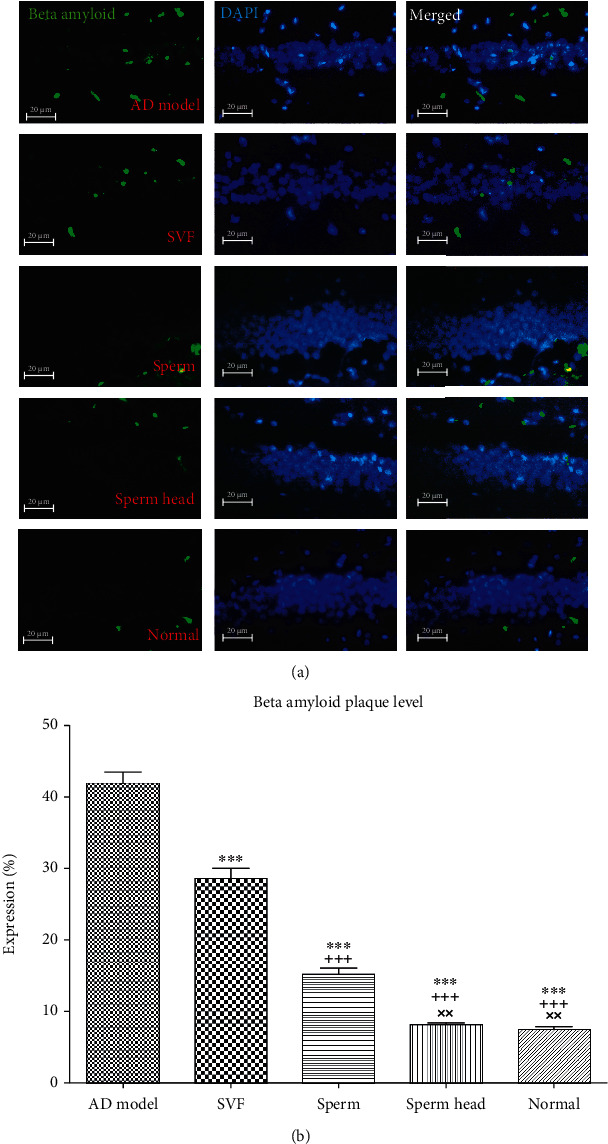


In our setting of AD animal model, A*β* plaques were significantly increased if compared to the normal animals and lasted for at least 21 days after the disease induction. We next examined how treatment of AD model with SVF, sperm, or sperm head affects A*β* plaque burden seven days after treatment (Figures 4(a) and 4(b)). Administration of SVF and sperm significantly decreased A*β* plaques comparing with the control AD model (*p* < 0.001). Nevertheless, A*β* plaques in SVF-treated AD model were significantly more than that of sperm-treated AD model (*p* < 0.001). Noteworthy to mention that just treatment using sperm head returned the level of A*β* plaque level back to the normal state (Figures 4(a) and 4(b)). A*β* plaques in sperm-treated AD model were significantly higher than that of sperm head-treated AD model and normal animals (*p* < 0.01). Considering the key role of brain lymphatic system in clearance of A*β* plaques [2, 3], we further evaluated if the changes in the brain lymphatic vessels are involved in lowering A*β* plaques level. In addition, given the important role of neural growth factors in brain tissue reconstitution [12, 15], the strength of SVF, sperm, or sperm head in stimulation of neural growth factor secretion was evaluated.

### 3.2. SVF, Sperm, and Sperm Head Differ in Their Potency to Restore Brain Lymphatic Vessels and Neural Growth Factors Levels

**Figure 5:**
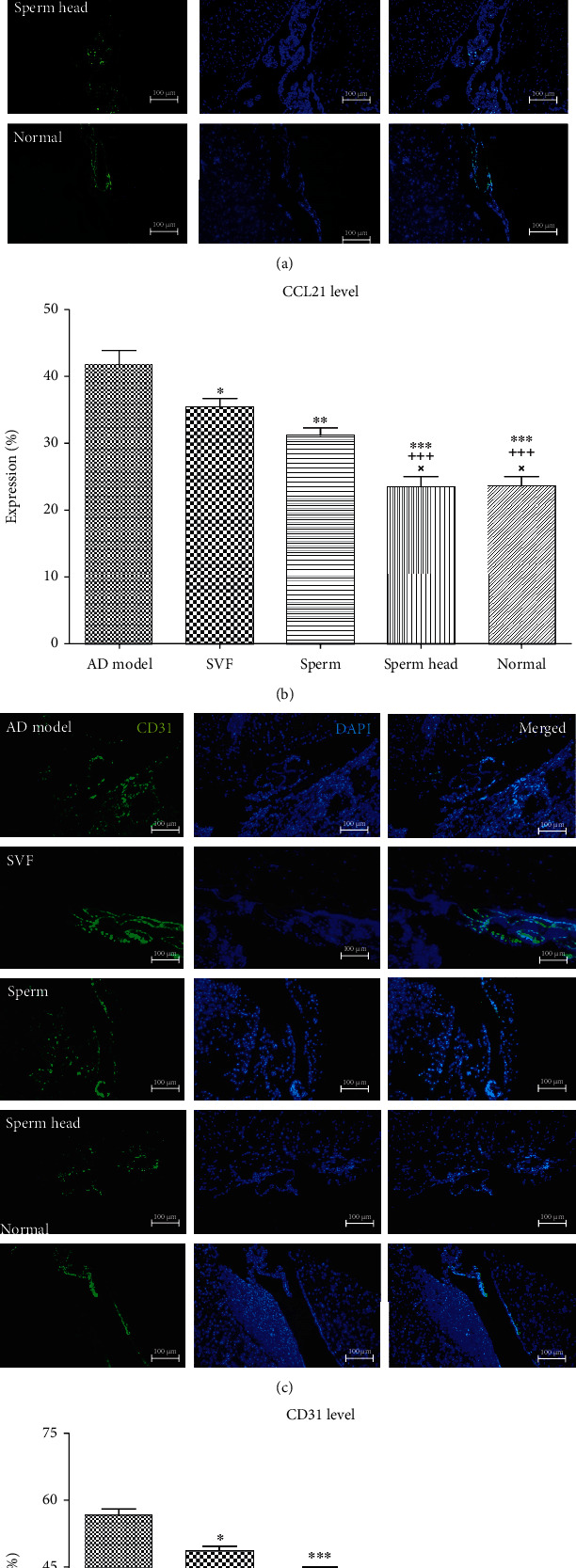


**Figure 6:**
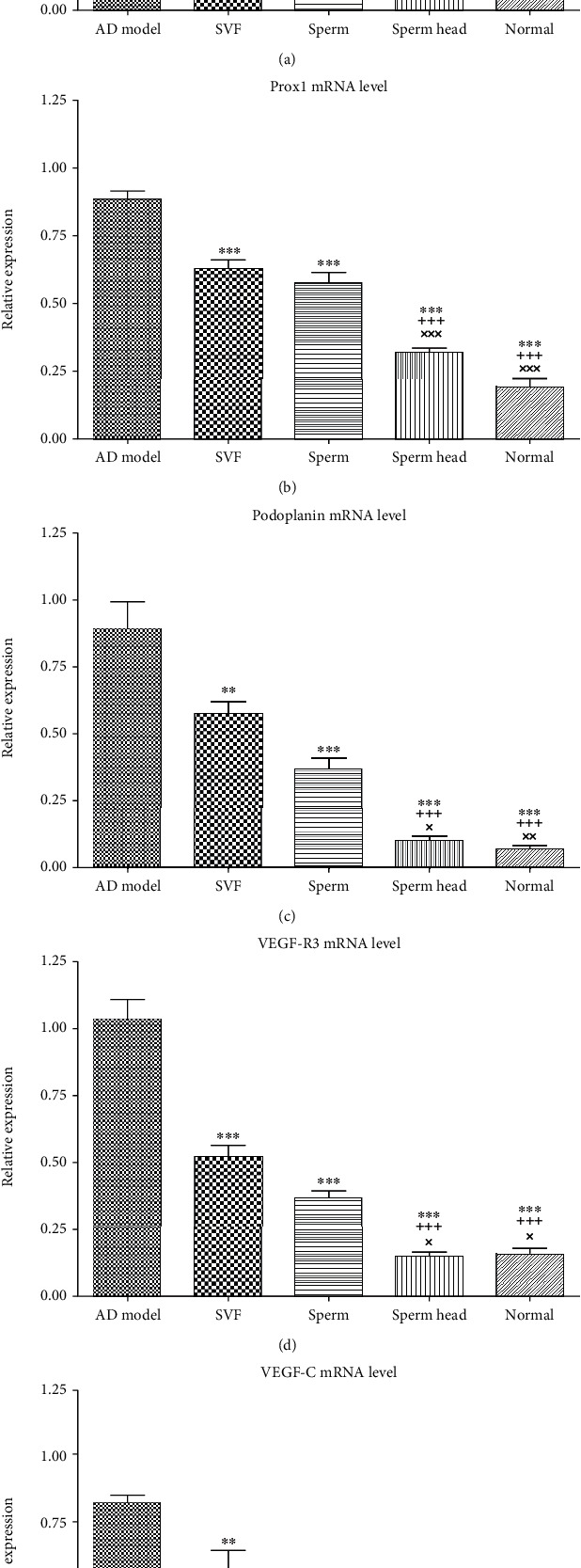


**Figure 7:**
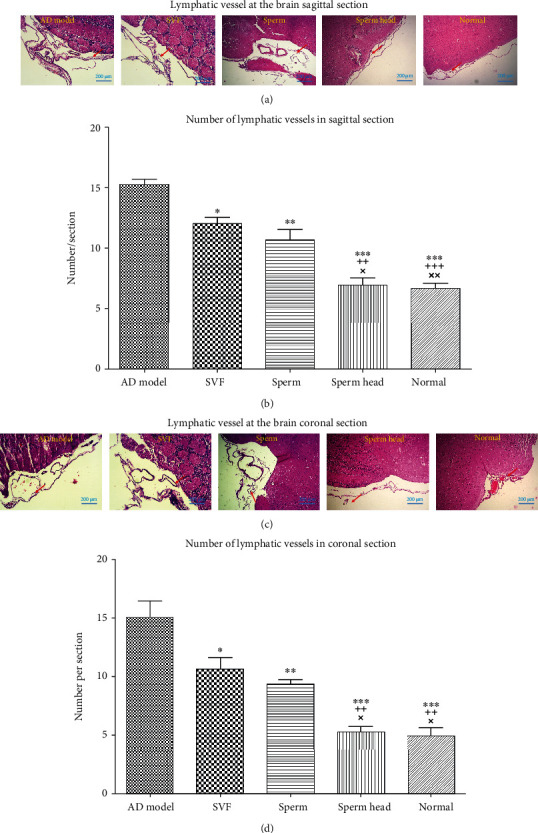


Lymphatic vessels along superior sagittal sinus were evaluated using CCL21, CD31, *LYVE-1*, *Prox1*, *PDPN*, and *VEGF-R3* [2, 3, 35, 36]. Immunohistochemical staining of the first two markers of the lymphatic vessels, i.e., CCL21 and CD31, showed that the two markers were significantly increased in the control AD models compared to the normal animals (*p* < 0.001, Figures 5(a)–5(d)). CCL21 and CD31 levels were significantly decreased in animals treated with SVF, sperm, or sperm head compared to the control AD model (*p* < 0.05, *p* < 0.01, *p* < 0.001). However, SVF- or sperm-treated animals showed significantly more CCL21 and CD31 levels than that of sperm head-treated AD model and normal animals (*p* < 0.001, *p* < 0.05). Treatment with sperm head led to normalization of CCL21 and CD31 levels as there was no significant difference between sperm head-treated animals with the normal group. Real-time PCR demonstrated that other markers of the brain lymphatic vessels including *LYVE-1*, *Prox1*, *PDPN*, and *VEGF-R3* were significantly increased in the control AD model compared to the normal group (*p* < 0.001, Figures 6(a)–6(d)). The level of mRNA corresponding to *LYVE-1*, *Prox1*, *PDPN*, and *VEGF-R3* molecules was significantly changed with different extent. Treatment with SVF significantly decreased expression of *LYVE-1*, *Prox1*, *PDPN*, and *VEGF-R3* comparing with the control AD model (*p* < 0.05, *p* < 0.01, *p* < 0.001, *p* < 0.001). In addition, treatment of AD model with sperm markedly lowered the mRNA level of *LYVE-1*, *Prox1*, *PDPN*, and *VEGF-R3* comparing with the control AD model (*p* < 0.001). Nevertheless, mRNA expression of *LYVE-1*, *Prox1*, *PDPN*, and *VEGF-R3* in SVF- or sperm-treated AD model was still significantly more than that of sperm head-treated AD model or normal animals (*p* < 0.001). Among the three types of therapy, only treatment by sperm head was statistically similar to normal group. To further estimate the changes in these vessels, H&E-stained tissue of sagittal and coronal sections of brain was also assessed. Lymphatic features were defined and characterized as vessels lined by flattened endothelial cells, in the absence of erythrocyte, and absence or presence of lymphocyte [30]. The lymphatics were evaluated around the cranial nerve and superior and inferior sagittal vessels in the entire tissue section. As shown in Figures 7(a)–7(d), brain lymphatic vessels in the animals treated with SVF, sperm, or sperm head were significantly decreased compared to the control AD model (*p* < 0.05, *p* < 0.01, *p* < 0.001). However, the level of lymphatics in SVF- or sperm-treated AD model was still markedly more than that of sperm head-treated AD model or normal animals (*p* < 0.01). Accordingly, mRNA level of the growth factor for lymphangiogenesis, i.e., *VEGF-C*, was also markedly up regulated [37] in the control AD model comparing with the normal group (*p* < 0.001) and attenuated in SVF-, sperm, or sperm head-treated AD models (*p* < 0.01, *p* < 0.001, *p* < 0.001). However, *VEGF-C* level on SVF- (*p* < 0.001) or sperm-treated AD model (*p* < 0.05) was significantly more than sperm head-treated AD model and normal animals. There was no significant difference in *VEGF-C* level between sperm head-treated AD model and normal animals. SVF or sperm were not potent enough to down-regulate the mRNA level of *VEGF-C* to normal level (Figure 6(e)).

**Figure 8:**
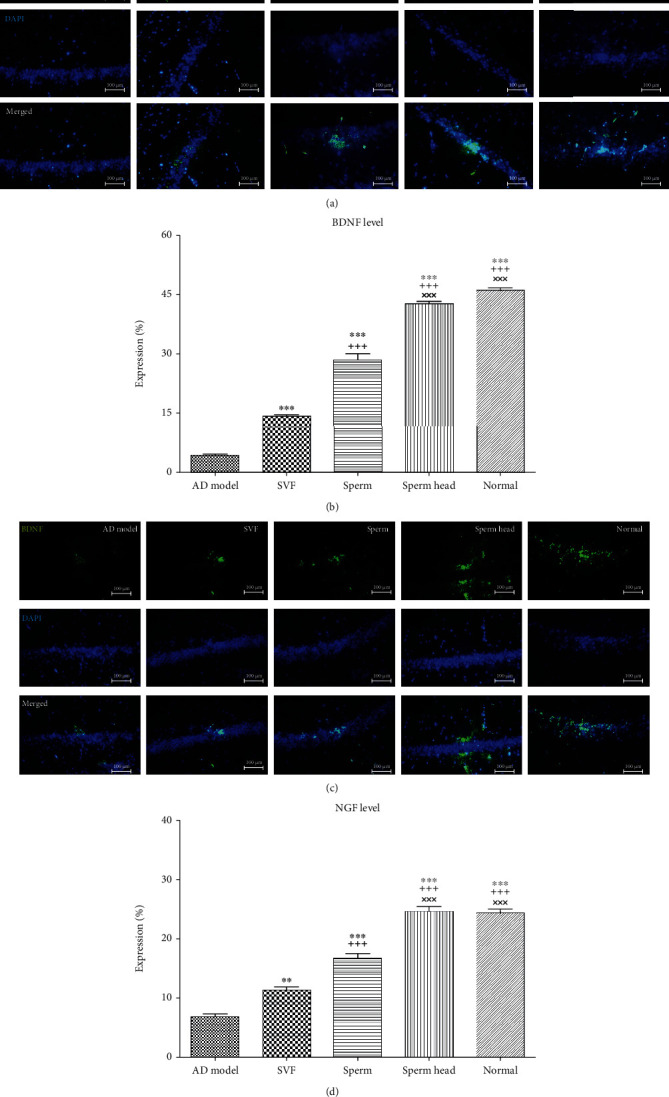


Malformation of lymphatics is associated with imbalance of neural growth factors level [2, 3, 14, 38]. On this basis, we also evaluated the level of neural growth factors which also decrease neural apoptosis and are involved in learning [39, 40]. As Figures 8(a)–8(d) show, BDNF and NGF levels were markedly decreased in hippocampus of AD model comparing with the normal group. Treatment with SVF, sperm, or sperm head led to a significant increase in BDNF and NGF level compared to the control AD model. However, treatment with sperm head was the most potent approach to significantly stimulate BDNF and NGF secretion comparing with SVF or sperm treatment.

Apart from abnormal removing process of A*β* plaques, the ongoing chronic inflammation in the brain is also another hallmark of Alzheimer's disease which plays important role in the development and pathological aspect of the disease [12, 41]. On this basis, microgliosis and astrogliosis as well as leukocyte trafficking and homing into the brain were then evaluated to see which one of the therapeutic approaches exerted in this study affects microglia and astrocytes inflammatory status and leukocyte trafficking in the inflamed brain affected by AD.

### 3.3. Effect of Treatment with SVF, Sperm, or Sperm Head on Microgliosis, Astrogliosis, and Trafficking of Leukocyte

**Figure 9:**
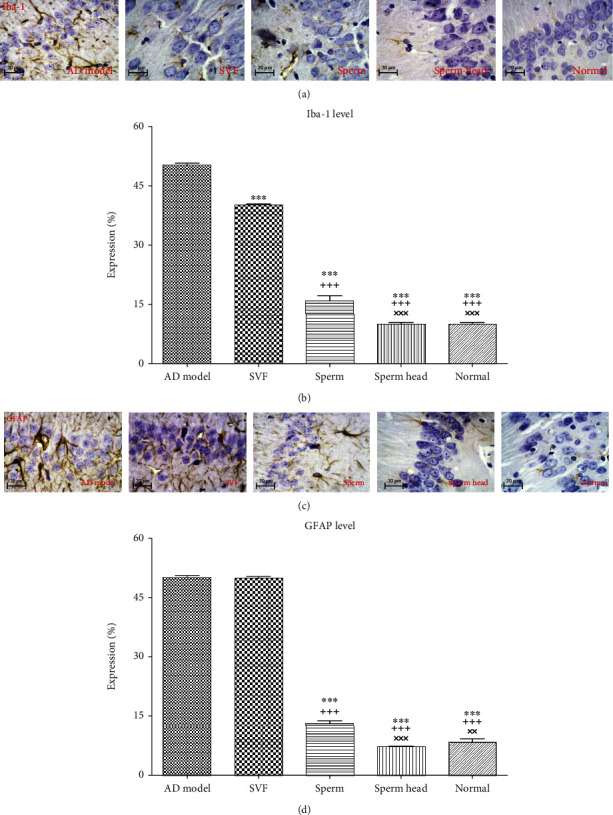


Microglia and astrocyte have pivotal role in modeling of brain microenvironment. Astrogliosis and microgliosis are hallmarks of chronic neuroinflammation and participate in mismodeling of brain microenvironment and AD pathology [4]. To find out how microgliosis and astrogliosis are affected after treatment, we also measured Iba-1 and GFAP which are known as microgliosis and astrogliosis markers, respectively [4, 42]. As Figures 9(a) and 9(b) demonstrate, treatment with SVF, sperm, and sperm head significantly decreased Iba-1 compared to the control AD model (*p* < 0.001). However, Iba-1 level in SVF-treated group was significantly higher than that of sperm-treated group (*p* < 0.001), sperm head-treated group (*p* < 0.001), and normal animals (*p* < 0.001). Notably, Iba-1 level in animals treated with sperm head was statistically similar to that of the normal state and was significantly lower than that of AD model, SVF-treated, and sperm-treated groups (*p* < 0.001). Figures 9(c) and 9(d) also indicate that administration of SVF did not affect GFAP level while sperm significantly decreased GFAP level comparing to the control AD model (*p* < 0.001) and SVF-treated animals (*p* < 0.001). Notably, treatment with sperm head was significantly lesser than that of the sperm-treated animals (*p* < 0.001) and lowered GFAP level down to the level of normal state.

**Figure 10:**
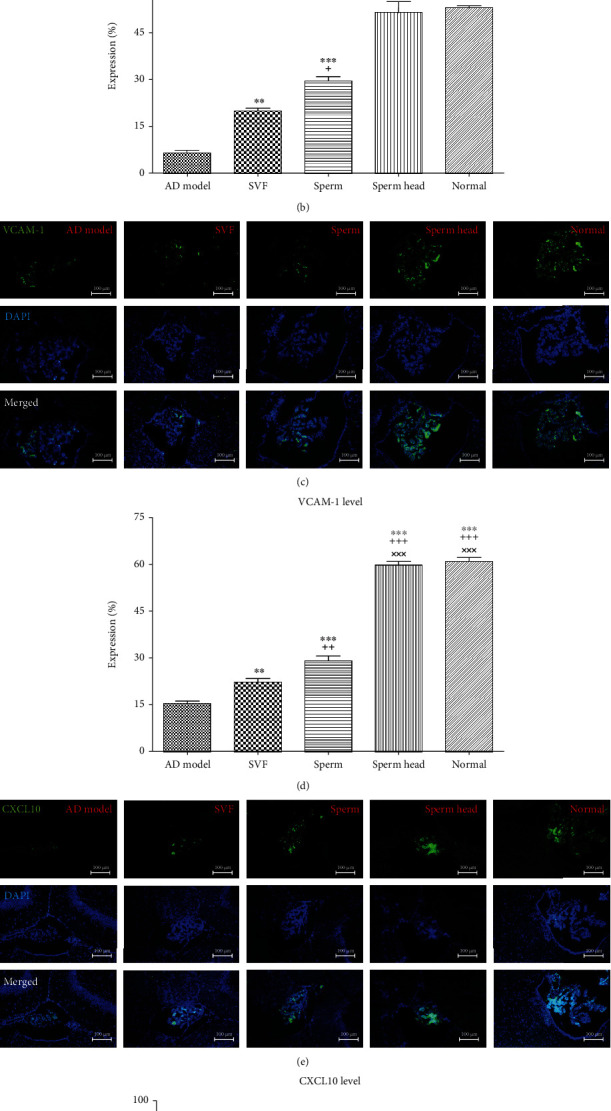


Abnormal choroid plexus gateway function is one of the hallmarks of AD. This is due to lower expression of leukocyte homing and trafficking markers, including ICAM-1, VCAM-1, and CXCL10 than normal animals [12]. We estimated the gateway function by evaluating the level of the three molecules in choroid plexus after ICV treatment of AD model with SVF, sperm, or sperm head and comparison was made with control AD model and normal animals. As demonstrated in Figures 10(a)–10(f), expression of the three molecules was decreased in the control AD model comparing with the normal group. While treatment with SVF led to significant increase in two of the three markers, i.e., ICAM-1 and VCAM-1 (*p* < 0.01) but treatment of AD model with sperm led to a marked increase in all of the three markers (*p* < 0.001). Nevertheless, treatment with sperm head was more potent than SVF (*p* < 0.001) and sperm (*p* < 0.05) and could increase all of the three markers up to normal level. SVF or sperm were not potent enough to normalize expression of the three molecules up to the normal level.

To reveal activation status of microglia, MHC II, CD86, and CD40, which are inflammatory markers and necessary to interact with brain-infiltrating CD4^+^ immune cells, were then analyzed [5, 9].

### 3.4. SVF, Sperm, and Head of Sperm Affect Inflammatory Markers of Microglial Cells

**Figure 11:**
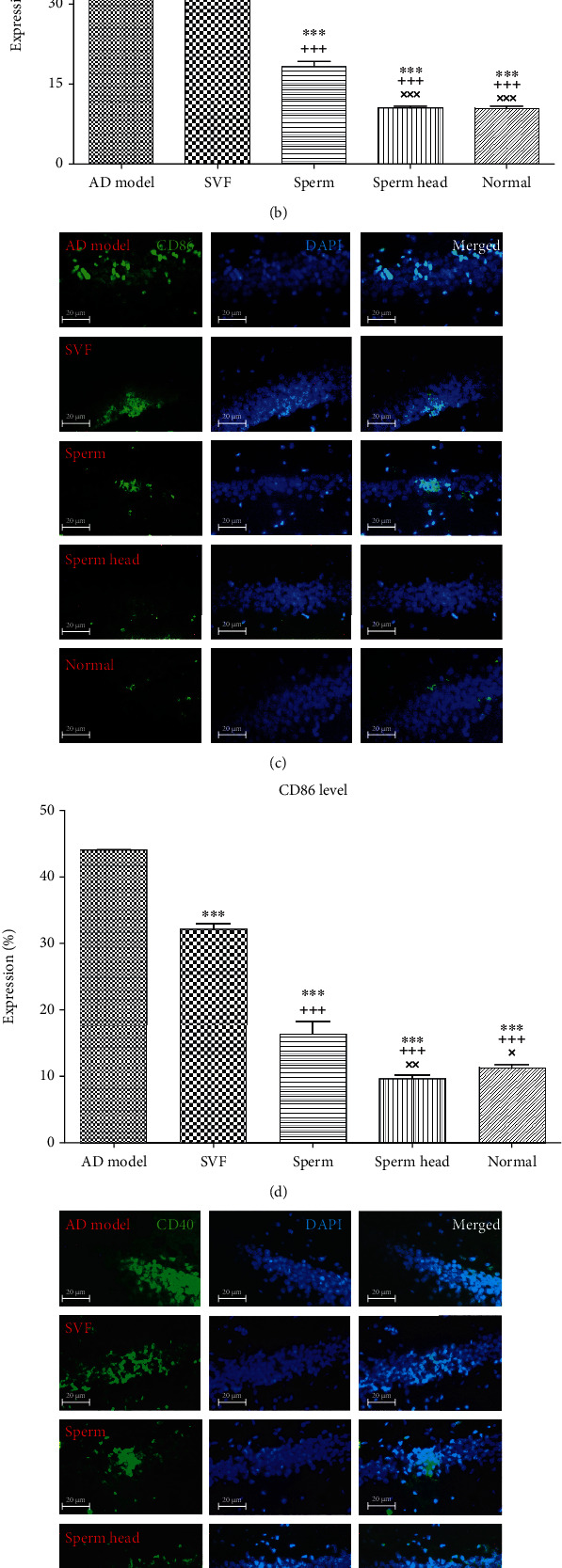


The expression of MHC II, CD86, and CD40 was in the order of control AD model>SVF>sperm>sperm head≈normal (Figure 11). On this basis, SVF-treated animals expressed significantly lower MHC II, CD86, and CD40 than that of the control AD model (*p* < 0.01). However, the level in SVF–treated animals was still markedly more than that of sperm- or sperm head-treated animals (*p* < 0.001). In addition, sperm-treated animals expressed significantly more MHC II (*p* < 0.001), CD86 (*p* < 0.01), and CD40 (*p* < 0.05) than that of the sperm head-treated animals. No significant difference was observed in MHC II, CD86, and CD40 levels between the sperm head-treated animals comparing with the normal group.

**Figure 12:**
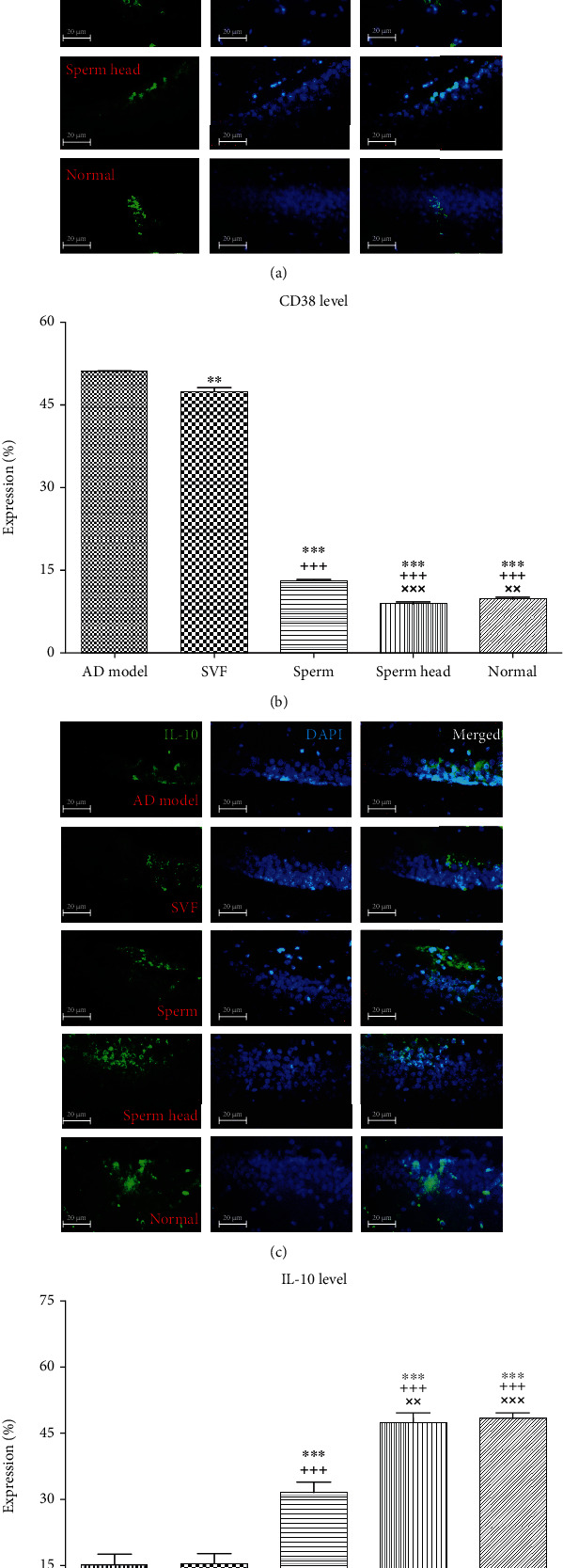


Microglia activation status was further evaluated based on CD38 and IL-10 markers. Given the role of CD38 in Alzheimer's pathology with important consequences for injury and repair processes in the brain and considering it as a marker of M1 macrophages [43], CD38 level was investigated after treatment with SVF, sperm, or head of sperm. Consistent with anti-inflammatory phenotype of microglia, CD38 expression was markedly attenuated in the animals treated with SVF compared to the control AD model group (*p* < 0.01, Figures 12(a) and 12(b)). In addition, sperm-treated group showed a decreased CD38 level comparing to the control AD model and SVF-treated groups (*p* < 0.001). Notably, treatment with sperm head returned CD38 level down to the level of normal state and was lower than the SVF-treated and sperm-treated group (*p* < 0.001).

Considering IL-10 as a marker of M2 macrophages with neuroprotective effects, expression of IL-10 was investigated [5, 44]. Treatment with SVF did not affect IL-10 level comparing to the control AD model. In line with anti-inflammatory phenotype of microglia, IL-10 expression was significantly increased in the animals treated with sperm or sperm head comparing to the control AD model and SVF-treated AD model (*p* < 0.001). However, IL-10 level in sperm-treated group was significantly lower than that of sperm head-treated and normal groups (*p* < 0.001). Notably, sperm head could increase IL-10 level up to the normal state (Figures 12(c) and 12(d)).

The ongoing chronic inflammation in the brain affected by AD [12, 39] caused by inflammatory microglia/astrocytes accumulating in high number near plaques [45], dysregulated leukocytes trafficking, A*β* and other toxicants accumulation, and abnormal lymphatic is associated with increase of Treg level and hypoxic condition [2, 3, 14, 38].

### 3.5. SVF, Sperm, and Sperm Head Differ in Their Potency to Reverse Treg Levels and Hypoxic Condition Back to the Normal State

**Figure 13:**
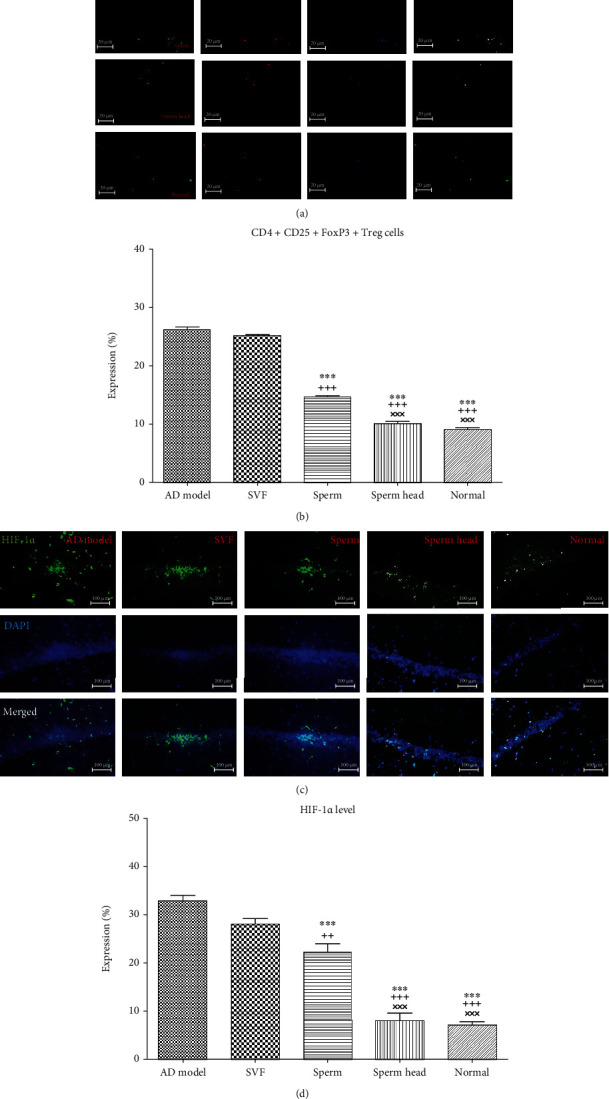


Tregs not only regulate immune response and establish tolerance but also are key players in resolving tissue inflammation and tissue healing [46]. The exact impact of Treg and its therapeutic effect in Alzheimer's disease have been a matter of ambiguity so far [17–20]. Therefore, Treg level was evaluated after administration of SVF, sperm, or head of sperm and compared with the control AD model and normal healthy animals. As Figures 13(a) and 13(b) show, there was no significant difference in Tregs between SVF-treated animals with the control AD model. Interestingly, treatment with intact sperm (*p* < 0.01) or sperm head (*p* < 0.001) significantly decreased Treg level compared to the control AD model or SVF-treated AD model. However, sperm-treated AD model still had significantly more Treg than that of the sperm head-treated AD model and normal animals (*p* < 0.05).

Hypoxia is a key element in Treg homeostasis and increases the potency of Tregs [47, 48] associated with neuroinflammation. Hypoxic response is mediated by HIF-1*α* [13]. The neuroinflammatory process ongoing in the brain associated with malformation of the lymphatics also leads to hypoxia [14]. To evaluate how treatment approaches applied in this study affects hypoxia, we also evaluated HIF-1*α* level in animals treated with SVF, sperm, or sperm head and compared with the control AD model and normal animals (Figures 13(c) and 13(d)). HIF-1*α* was significantly increased in AD model comparing with the normal animals as detected by immunohistochemical staining of brain tissue sections (*p* < 0.001) and treatment with SVF did not change HIF-1*α* level. Inversely, treatment with sperm or sperm head led to a significant decrease in HIF-1*α* level compared to the control AD model (*p* < 0.001) or SVF-treated AD model (*p* < 0.001). Notably, treatment with sperm head was significantly lower than that of sperm-treated AD model (*p* < 0.001) and potent enough to reverse hypoxic condition in the hippocampus.

## 4. Discussion

In this study, we utilized and compared two approaches of suppressing or boosting inflammatory process. Inspired by the results of reproductive immunology [22–25], this was done by simulation of immunologic properties of SVF and sperm in the brain. The results demonstrated that sperm with natural inflammatory effect has therapeutic potency in animal model of Alzheimer's disease. The therapeutic capacity of sperm became optimized when its head was applied. Sperm head could resolve chronic neuroinflammation in the diseased brain and repaired it backing into normal condition. Sperm head with natural inflammatory property acted much more potent than SVF with natural anti-inflammatory effect. In other words, anti-inflammatory strategy did not lead to resolution of inflammation and modulation of damaged brain structure and function. Our results suggest that the second approach, i.e., boosting inflammatory approach using sperm, works better for the resolution of inflammation in the brain of an animal model of AD than anti-inflammatory strategy. This may seem a confusing matter. However, to clarify the figure, there is important notion regarding inflammatory response of the immune system that deserve to be discussed.

The immune system has a central role in tissue repair and regeneration as it is crucial in determining the speed and the outcome of the healing process. The healing process entails three distinct yet overlapping including inflammation, cell proliferation and new tissue formation, and remodeling and maturation. Considered the initial stage of healing, inflammation has important role in determining the extent of scarring and the restoration of organ function [49, 50]. Two flavors of inflammation have been described, including constructive or destructive. If tissue integrity and homeostasis are restored, constructive inflammation finally resolves ending with repair. Inversely, if the healing process is not properly regulated, chronic inflammatory process persists, impairs normal tissue function, and ultimately leads to organ failure. Therefore, it is the inflammatory process which is a prerequisite stage and activates tissue repair and regeneration. Not surprisingly, the molecular mechanisms and signaling pathways that control repair and regeneration are interlinked with those that control inflammatory process [49, 51]. Immune cells are involved from the inflammatory phase to the healing and regeneration phase. They establish communication with tissue-resident cells to restore normal tissue structure and function by clearing tissue debris, normalization of vasculature, and supporting regeneration of parenchymal cells. It has become evident that the type and profile of immune cells present at the site of injury have powerful influences on the other cells involved in the quality of tissue repair, regeneration, and remodeling [50, 52, 53]. Recent therapies are now focusing on modulation of immune-mediated mechanisms of tissue repair and regeneration. The goal of these therapies is to restore tissues and whole organ systems back to a normal or highly functional state. These approaches rely on modulating the inflammatory cells that affect the terrain for subsequent steps of tissue repair [50, 53]. For example, a report suggested depletion of Treg which has the danger of autoimmunity [20], while we have not observed such a danger by the approach proposed in this article by now. On this basis for devising a successful approach, a deep understanding of how complicated network of inflammatory process is initiated, regulated, and limit or allow damaged tissue normalization is required. Given the paucity of our knowledge about the precise mechanism of inflammation and tissue repair, we proposed a nature-inspired approach of cell therapy based on reproductive immunology. The results demonstrate that sperm had intrinsic property to shift the inflammatory process towards a constructive inflammation. Based on our previous study [27], sperm head programs neutrophil in a way enabling them to direct inflammatory process towards constructive mode. The beneficial role of constructive inflammation was previously shown after a traumatic injury to the spinal cord [54, 55]. Reparative function of inflammation in normal hippocampal neurogenesis and remyelination has also been shown to impair in mice devoid of T cells and macrophages [16]. Indeed, the dynamic interactions between various cell types during inflammatory process have determinant effect on the fate of the tissue affected leading to repair and regeneration or more damage [50, 52, 56, 57]. Therefore, the old insight towards inflammation considered only “good” or “bad” is no longer helpful to devise immunotherapeutic approach [56, 58].

We focused on lymphatic vessels because of their role in multiple homeostatic functions such as purge of toxicants and immune surveillance. Lymphangiogenesis increases during inflammation and returns back to normal level after physiologic repair [59]. Immune cells are able to orchestrate lymphangiogenesis in inflamed brain and sperm head causes recruitment of the immune cells required for physiologic repair. Normalization of lymphatics is a new therapeutic approach for activating repair physiologic mechanism resolution to resolve chronic inflammation [3] which has not been achieved so far. Lessons learnt from other chronic inflammatory diseases like tumor immunology [16] better clarify the importance of vascular normalization and just increasing or decreasing vascular level would not help repair process [60]. Lymphatics also play a crucial role in regulating the inflammatory response by influencing drainage of toxicants, inflammatory mediators, leukocytes trafficking, and homing of immune cells into CNS which are also dysregulated in Alzheimer's. This phenomenon is also similarly observed in tumor in which anti-inflammatory cells are recruited and inhibit resolution of inflammation [20, 55, 61]. Normalization of lymphatics occurred along with leukocyte trafficking across choroid plexus gateway and decrease of hypoxia. Notably, we observed that decrease of hypoxia was associated with modulation of Treg level and decrease of microgliosis, astrogliosis, and inflammatory markers.

Tregs maintain immune tolerance and perform specialized functions in tissue homeostasis and remodeling [46]. Under normal conditions, Tregs attend in the brain in a low number but they massively accumulate in neuroinflammatory condition with the aim of resolution of inflammation and recovery [62]. However, in case of AD, Tregs increase in the brain and their role in neurological recovery is controversial [17–20]. Based on similarities between tumor and AD, such as hypoxia, Tregs accumulation may even worsen the condition in AD [19, 20]. Previous studies via immune-suppressive approaches such as corticosteroids, metformin, angiotensin type II receptor blocker, or anti-oxidant were not also successful in full recovery of AD and had just prophylactic effects [4, 63–66]. The reason may lie beneath the fact that immunosuppressive strategy does not decrease Treg levels, inhibit boosting inflammation, and therefore does not efficiently modulate glial cell inflammatory status [20]. Similarly, the relationship between glial cells activation status evaluated using MHC II, CD86, and CD40 with Treg levels was previously shown in animal model of multiple sclerosis before [9]. Other studies applying Treg stimulation had just ameliorating effects and did not lead to complete resolution of neuroinflammation [17, 18]. Presumably, Tregs are the main obstacles as they inhibit inflammatory response and so the inflammatory response does not perform its natural pathway ending up with repair and regeneration. Based on the previous report, similar to tumor, Tregs exert inhibitory effects on trafficking of immune cells into CNS preventing resolution of inflammation and repair [20, 61]. Consistently, sperm head could restore choroid plexus gateway function but not SVF. On the other hand, hypoxia, which is also as an attractant factor for Tregs, was markedly decreased.

Consistent with Treg level and hypoxia along with the balancing effect of IL-10 in the brain [44], promotion of inflammation using sperm head made microglia switch from M1 to M2 phenotype, represented by CD38 and IL-10, respectively [43, 44]. The M2 microglia phenotype collaborating in the constructive inflammatory process observed in this study [51, 56, 58] is consistent with the reparative effects. Inversely, anti-inflammatory strategy based on SVF could not make changes as efficient as sperm head did on microglia and astrocyte inflammatory status, hypoxia, and Treg levels.

Anti-inflammatory strategy by SVF could only partially change inflammatory status of microglia and astrocytes. However, boosting inflammation using sperm head could modulate inflammatory status of microglia and astrocytes to desired level affect Treg levels and decrease hypoxia. Such an inflammatory reaction run by sperm head can be regarded as a constructive inflammatory process as discussed before [27, 51, 56, 58]. The results of this study demonstrate that sperm head decreases Tregs and hypoxia promoting constructive inflammatory process ending up with repair in any organ other than the uterus during mating [67].

Traditional strategies in regenerative medicine based on stem cells, growth factors, and/or biomaterials have not yet proven broadly effective in the clinic. Genetic basis is an important obstacle in stem cell therapy. In addition, the regenerative potency of stem cells is influenced by local immune cells orchestrating network of tissue damage [50, 52]. Alternatively, sperm head has natural potency to turn the destructive inflammation towards constructive inflammation and normalize the damaged brain. Presumably, sperm head constituents are familiar for immune system acting like a secret code for immune system. Being observed by immune cells, they reset the inflammatory process and turn the tissue towards its default natural architecture. This approach does not depend on genetic basis and interestingly works better when applied in allogeneic setting. As an important notable safety issue, the basis of this approach is based on the natural process of mating which hints the safety of this method.

## 5. Limitations

The animal model of AD utilized in this study had similarities with the human AD disease such as features of impairment in behavioral response, chronic inflammation, neurotoxic consequences involving gliosis, and structural and microenvironmental abnormalities [1–3, 12–15]. Intra-hippocampal injection of A*β* initiates an acute inflammation which transits to a chronic inflammatory process within one week after A*β* injection. The models based on A*β* injection are known to replicate the changes in cellular properties and brain microenvironments evident in inflamed brain in the progression of AD pathology [68, 69]. However, sudden changes made within short time may be a limitation of the models set up by injecting A*β* including the model used in this study. It is likely that activation of microglia can potentially lead to functional cell responses which confer neuroprotection [68]. Nevertheless, the lifespan of rats is short (~4 years) and this can compensate for the effect of sudden changes made following beta-amyloid injection. Another limitation with animal model of AD is species-dependent variations in the damage produced in the brain [68, 69]. As for needle-mediated inflammation, it has been proposed that control animals receiving saline injection may also exhibit low relatively small extents of microgliosis [70]. To prevent such errors, in this study, two saline-injected controls were considered including 1) normal animals receiving saline in hippocampal region and 2) AD model animals receiving saline via ICV route. The former group receiving saline in hippocampal region did not develop AD pathological signs observed in the animals receiving A*β* in saline. In addition, the latter group included AD model animals receiving saline via ICV route that did not show therapeutic effects.

The results of the animal model can be extended to human as an approximate equivalent effective dose for administration to human cases can be calculated using known formulas [71, 72].

It is noteworthy to mention that we observed similar therapeutic efficacy using sperm head from different species, e.g., human sperm head had similar therapeutic effect on rat AD model (article under preparation). In addition, non-invasive application of this approach via mucosal route, i.e., nasal or rectal, for the brain and other peripheral organs, which has been examined in our laboratory, facilitated application of this approach for clinical use in other chronic inflammatory diseases (unpublished data). More pilot studies on a few animal model of chronic inflammatory disease have been performed with promising results.

## 6. Conclusion

This study for the first time shows the intrinsic properties of sperm head to shift the chronic inflammation and deviate towards a constructive inflammatory process in organs other than the reproductive system. Results propose sperm head as a new paradigm of cell therapy. Promotion of constructive inflammation by sperm head in the brain is similar to the process occurred in the uterus during mating. On this basis, modulation, but not suppression, of inflammatory process helps resolve neuroinflammation and repair damaged tissue. Given the limited regeneration capacity of the brain, it is conceivable to extend this approach to other damaged organs. This proposal is based on the immunologic properties of sperm head that has been observed in the uterus and brain. The results of the animal model can ideally be translated to human cases.

## Data Availability

The data that support the findings of this study are available from the corresponding author, Dr. Nafiseh Pakravan, upon reasonable request.

## Abbreviations

AD: Alzheimer's disease
A*β*: Amyloid-*β*
BDNF: Brain-derived neurotrophic factor
CNS: Central nervous system
CCL21: Chemokine (C-C motif) ligand 21
CD4: Cluster of designation 4
CD25: Cluster of designation 25
CD31: Cluster of designation 31
CD38: Cluster of designation 38
CD40: Cluster of designation 40
CXCL10: C-X-C motif chemokine-10
DAPI: 4 ′-6-diamidino-2-phenylindole
DAB: 3, 3′-diaminobenzidine
FOXP3: Forkhead box protein 3
GFAP: Glial fibrillary acidic protein
GAPDH: Glyceraldehyde 3-phosphate dehydrogenase
H&E: Haematoxylin and eosin
HIF-1*α*: Hypoxia inducible factor-1*α*
Iba-1: Ionized calcium-binding adaptor molecule-1
IL-10: Interleukin-10
ICV: Intra-cerebro-ventricular
ICAM-1: Intercellular adhesion molecule-1
LYVE-1: Lymphatic vessel endothelial hyaluronan receptor-1
MHC II: Major histocompatibility complex II
NGF: Nerve growth factor
PBS: Phosphate buffer saline
PDPN: Podoplanin
Prox1: Prospero-related homeobox1
Treg: Regulatory T cell
SVF: Seminal vesicle fluid
VCAM-1: Vascular cell adhesion molecule-1
VEGF-R3: Vascular endothelial growth factor receptor 3.
